# Vulnerable combinations of functional dopaminergic polymorphisms to late-onset treatment resistant schizophrenia

**DOI:** 10.1371/journal.pone.0207133

**Published:** 2018-11-08

**Authors:** Kengo Oishi, Nobuhisa Kanahara, Masayuki Takase, Yasunori Oda, Yusuke Nakata, Tomihisa Niitsu, Masatomo Ishikawa, Yasunori Sato, Masaomi Iyo

**Affiliations:** 1 Department of Psychiatry, Chiba University Graduate School of Medicine, Chuou-ku, Chiba, Chiba, Japan; 2 Division of Medical Treatment and Rehabilitation, Chiba University Center for Forensic Mental Health, Chuou-ku, Chiba, Chiba, Japan; 3 Zucker Hillside Hospital, Glen Oaks, NY, United States of America; 4 Department of Global Clinical Research, Chiba University Graduate School of Medicine, Chuou-ku, Chiba, Chiba, Japan; Hudson Institute, AUSTRALIA

## Abstract

**Background:**

A significant portion of patients with schizophrenia who respond to initial antipsychotic treatment acquire treatment resistance. One of the possible pathogeneses of treatment-resistant schizophrenia (TRS) is antipsychotic-induced dopamine supersensitivity psychosis (Ai-DSP). Patients with this disease progression might share some genetic vulnerabilities, and thus determining individuals with higher risks of developing Ai-DSP could contribute to preventing iatrogenic development of TRS. Therefore, we decided to examine whether combinations of functional single nucleotide polymorphisms (SNPs) known to affect dopaminergic functions are related to Ai-DSP development.

**Methods:**

In this case-control study, 357 Japanese participants diagnosed with schizophrenia or schizoaffective disorder were recruited and divided into two groups, those with and without Ai-DSP. As functional SNPs, we examined rs10770141 of the tyrosine hydroxylase gene, rs4680 of the catechol-O-methyltransferase gene, and rs1799732 and rs1800497 of the *DRD2* genes, which are known to possess strong directional ties to dopamine synthesis, dopamine degradation and post-synaptic DRD2 prevalence, respectively.

**Results:**

Among the 357 Japanese patients with schizophrenia or schizoaffective disorder, 130 were classified as Ai-DSP(+) and the other 227 as Ai-DSP(-). Significantly higher proportions of Ai-DSP(+) patients were found to have the SNP combinations of rs10770141/rs4680 (57.9%, OR2.654, 95%CI1.036–6.787, P = 0.048) and rs10770141/rs4680/ rs1800497 (64.3%, OR4.230, 95%CI1.306–13.619, P = 0.029). However, no single SNP was associated with Ai-DSP.

**Conclusions:**

We preliminarily found that carrying particular combinations of functional SNPs, which are related to relatively higher dopamine synthesis and dopamine degradation and lower naïve DRD2, might indicate vulnerability to development of Ai-DSP. However, further studies are needed to validate the present results.

## Introduction

Although 75% of first-episode schizophrenia patients positively respond to early antipsychotic medications [1, 2], a significant portion of them have been reported to become unresponsive to antipsychotics during pharmacotherapy [3–8]. One of the assumed pathogeneses of this late-onset treatment-resistant schizophrenia (TRS) is antipsychotic-induced dopamine supersensitivity psychosis (Ai-DSP), which may be associated with compensatory upregulation of dopamine D2 receptors (DRD2) due to over-blockade by antipsychotics [9–12]. It will be necessary to identify risk factors and prevention and treatment strategies for Ai-DSP in order to improve the outcomes of patients with schizophrenia [13].

DRD2 up-regulation is caused not only by blockade of DRD2 but also by dopamine depletion [14], suggesting its pathogenic complexity of Ai-DSP. There are various functional polymorphisms of genes associated with dopamine synthesis [15], dopamine degradation [16,17] and binding sites including DRD2 [18–21]. It has also been reported that multilocus genetic profiles of dopaminergic signaling might modulate the function of the striatum and working memory in schizophrenic patients [22]. Hence, we conjectured that these polymorphisms may determine the individual characteristics of dopaminergic function and affect psychotic symptoms, responses to antipsychotics and/or development of Ai-DSP.

Therefore, we conducted this first preliminary study to evaluate whether particular combinations of dopaminergic functional SNPs are associated with the development of Ai-DSP in patients with schizophrenia. In this study, we examined rs10770141 of the tyrosine hydroxylase (*TH*) gene [15], rs4680 of the catechol-O-methyltransferase (*COMT)* gene [16], and rs1799732 [18] and rs1800497 [19] of the *DRD2* gene. They are all functional SNPs, which have been reported to modify dopamine synthesis, dopamine degradation and dopamine receptors, respectively.

## Materials and methods

### Subjects

This case-control study included 357 patients who met DSM IV-TR criteria for schizophrenia or schizoaffective disorder. All the participants were Japanese and were seen at Chiba University Hospital or affiliated hospitals in the period from May 2001 to October 2014. The study was approved by the Ethics Committee of the Chiba University Graduate School of Medicine and was performed in accord with the Helsinki Declaration. We obtained blood samples from all patients who provided written informed consent for the use of their DNA. The capability of patients to give informed consent was evaluated by the treating psychiatrist. Any patients who lacked adequate understanding of the study were excluded.

### Assessment of dopamine supersensitivity psychosis

We classified the patients with schizophrenia into two groups, i.e., with or without Ai-DSP episodes, by investigating their medical records. To determine the presence or absence of Ai-DSP episodes, we followed previously used criteria [23,24]. Specifically, patients with any of the following three conditions were considered as Ai-DSP(+): 1) *Withdrawal psychosis*, an acute relapse or exacerbation of psychosis appearing within six weeks (oral medication) or three months (long-acting intramuscular injection) after dose reduction or discontinuation of antipsychotics; 2) *Development of tolerance to antipsychotics*, an acute relapse or exacerbation of psychosis, occurring independently of antipsychotic medication adjustment and remaining uncontrolled even by an increase in current antipsychotic dosages of 20%; and/or 3) *Tardive dyskinesia*. Participants who presented none of these conditions were classified as Ai-DSP(-).

Because Ai-DSP is, by definition, an iatrogenic consequence of antipsychotic treatment, we excluded patients experiencing their first psychotic episode. Hence, this study included only patients who had received at least one year of pharmacotherapy. In addition, the presence of comorbidities, such as substance abuse or dependencies, were also exclusion criteria.

### Single nucleotide polymorphisms and genotyping

We selected four SNPs to examine: rs10770141, rs4680, rs1799732 and rs1800497. Patients carrying the minor allele of rs10770141, i.e., T(+), have been reported to exhibit 30%-40% higher gene expression of *TH* compared to their T(-) counterparts [15]. rs4680 is a non-synonymous SNP, which causes the replacement of valine with methionine at residue 158 of COMT, resulting in almost 50%-75% reduction of the enzymatic activity of COMT [16]. Carriers and non-carriers of the methionine allele are expressed as Met(+) and Met(-), respectively. rs1799732, also known as -141C Insertion/Deletion and presented as Del(-) if the polymorphism is not present and Del(+) if it is, has been demonstrated to lower the gene expression of *DRD2* by 40% [18]. For rs1800497, imaging analyses by positron emission tomography revealed that individuals with the minor allele, i.e. A1(+), had 12% less availability of striatal DRD2 than those with the major allele, A1(-) [19].

For the determination of risk alleles, we considered how dynamic the concentration of dopamine at the synaptic cleft changes. Because higher dopamine synthesis and its degradation may lead to relatively rapid changes of the dopamine concentration at the synaptic cleft, we determined that T(+) of rs10770141 and Met(-) of rs4680 were risks. For DRD2, subjects with naïve lower density, i.e., Del(+) of rs1799732 and A1(+) of rs1800497, may need relatively lower doses of antipsychotics. However, they could also be at risk of over-blocking DRD2 because antipsychotics have a significantly longer half-life compared to endogenously released dopamine.

Whole blood samples were obtained from all the participants. A QIAamp DNA Blood Mini kit (250) (Qiagen, Valencia, CA) was used to isolate genomic DNA. Genotypes were determined by using TaqMan probe assays (Applied Biosystems, Foster City, CA) and ABI PRISM 7300 Sequence Detection System (Applied Biosystems). In order to perform all the procedures, we followed the protocols described in the manufacturer’s manuals. All polymerase chain reactions were conducted by one cycle at 95°C for 10 minutes, followed by 40 cycles (rs10770141, rs4680 and rs1800497) or 60 cycles (rs1799732) at 92°C for 15 seconds and 60°C for 60 seconds.

### Quality assessments

The quality of the study was evaluated by following the checklist of the Strengthening the Reporting of Genetic Association (STREGA) statement [25].

### Statistical analysis

We applied Student’s *t*-test and Fisher’s exact test for continuous and categorical variables, respectively. For combined SNP analyses, the patients with risk allele(s) for developing Ai-DSP were compared to those lacking the risk allele(s). The risk alleles for rs10770141, rs4680, rs1799732 and rs1800497 were considered to be T(+), Met(-), Del(+) and A1(+), respectively. The 95% confidential intervals were estimated by Fisher’s exact test. All statistical analyses were conducted with SPSS version 19.0 (IBM, Armonk, NY). The threshold for statistical significance was set at *P*<0.05.

## Results

### Comparisons of study groups

Clinical characteristics of the participants are summarized in Table 1. A total of 357 schizophrenia cases, consisting of 130 Ai-DSP(+) and 227 Ai-DSP(-) patients, were examined. The following data were missing: the genotypes of rs4680 for 2 Ai-DSP(+) and 3 Ai-DSP(-) patients and the chlorpromazine equivalent (CP-eq) doses of antipsychotics for 4 AiI-DSP(+) and 8 Ai-DSP(-) patients. There were no significant distributional differences in sex or age among Ai-DSP(+) patients [59 males and 71 females; mean age 52.1 years (standard deviation, SD = 15.2), range 21‒86 years] and Ai-DSP(-) patients [116 males and 111 females; average age 49.6 years (SD = 15.5), range 16‒92 years]. With respect to antipsychotic dosages, Ai-DSP(+) patients were found to be taking greater amounts [718.8 (SD = 448.6) CP-eq mg] than Ai-DSP(-) patients [519.6 (SD = 389.9) CP-eq mg]. Ai-DSP(+) patients also presented longer disease and treatment durations [28.7 (SD = 14.7) and 27.6 (SD = 15.2) years, respectively] than Ai-DSP(-) patients [23.2 (SD = 15.5) and 22.3 (SD = 15.5) years, respectively].

**Table 1:** Clinical characteristics of patients with schizophrenia.

	Schizophrenia cases						*P* values
	Total (*N* = 357)		AI-DSP (*N* = 130)		Non-AI-DSP (*N* = 227)		
Sex, male / female	175 / 182		59 / 71		116 / 111		N.S.^a^
Age, mean (SD), years	50.5	(15.4)	52.1	(15.2)	49.6	(15.5)	N.S.^b^
Age range, years	16–92		21–86		16–92		—
CP equivalent dose, mean (SD), mg	593.6	(423.2)	718.0	(448.6)	519.6	(389.9)	0.000^b^
Disease duration, mean (SD), years	25.3	(15.4)	28.7	(14.7)	23.2	(15.5)	0.001^b^
Treatment duration, mean (SD), years	24.3	(15.6)	27.6	(15.2)	22.3	(15.5)	0.002^b^

Abbreviations: AI-DSP, dopamine supersensitivity psychosis; CP, chlorpromazine; N.S., not significant

^a^ Comparisons between two groups were performed using the chi-square test.

^b^ Comparisons between two groups were performed using Student’s *t*-test.

### Single SNP analyses

The numbers and proportions of Ai-DSP(+) patients among selected SNP groups ranged from 19 to 111 and from 34.0% to 46.3%, respectively. The smallest population, but the highest proportion, of Ai-DSP(+) was observed in T(+): 19 Ai-DSP(+) patients, comprising 46.3%. T(-) patients had the largest Ai-DSP(+) population of 111. The lowest rate of Ai-DSP(+), 34.0%, was observed in Met(+) patients. We failed to obtain the genotype of rs4680 from 5 individuals (1.4% of 357 participants): 2 Ai-DSP patients (1.5% of 130) and 3 Non-Ai-DSP (1.3% of 227). All the genetic distributions were in Hardy-Weinberg equilibrium, as even the largest dissociation was 3.1% (S1 Table). None of these single allelic or genotyping distributions showed associations with developing Ai-DSP. The statistics for each allelic status, including the proportions of Ai-DSP(+) and odds ratios (OR), are shown in Table 2.

**Table 2:** Result summary of statistical analyses for all patient categories.

SNP ID	Subjects	Allele (%)				Proportion of AI-DSP(+), %		OR		95% Confidential Interval		*P* value^a^
		Risk		Protective		Risk	Protective			Lower	Upper	
Single SNP												
rs10770141		T(+)		T(-)		T(+)	T(-)					
	AI-DSP	19	(11.5)	111	(88.5)	46.3	35.1	1.595		0.834	3.050	0.171
	Non-AI-DSP	22		205								
rs4680		Met(-)		Met(+)		Met(-)	Met(+)					
	AI-DSP	64	(46.6)	64	(53.4)	39.0	34.0	1.240		0.803	1.914	0.375
	Non-AI-DSP	100		124								
rs1800497		A1(+)		A1(-)		A1(+)	A1(-)					
	AI-DSP	76	(58.3)	54	(41.7)	36.5	36.2	1.013		0.655	1.567	1.000
	Non-AI-DSP	132		95								
rs1799732		Del(+)		Del(-)		Del(+)	Del(-)					
	AI-DSP	44	(31.4)	86	(68.6)	39.3	35.1	1.196		0.756	1.894	0.478
	Non-AI-DSP	68		159								
Double SNP												
rs10770141; rs4680		T(+);Met(-)		T(-);Met(+)		T(+);Met(-)	T(-);Met(+)					
	AI-DSP	11	(5.4)	57	(47.4)	57.9	34.1	2.654		1.036	6.787	**0.048**
	Non-AI-DSP	8		110								
rs10770141; rs1800497		T(+);A1(+)		T(-); A1(-)		T(+);A1(+)	T(-); A1(-)					
	AI-DSP	14	(7.6)	49	(37.8)	51.9	36.3	1.890		0.833	4.288	0.137
	Non-AI-DSP	13		86								
rs10770141; rs1799732		T(+);Del(+)		T(-); Del (-)		T(+);Del(+)	T(-); Del (-)					
	AI-DSP	7	(4.2)	74	(61.3)	46.7	33.8	1.715		0.621	4.738	0.401
	Non-AI-DSP	8		145								
rs4680; rs1800497		Met(-); A1(+)		Met(+); A1(-)		Met(-); A1(+)	Met(+); A1(-)					
	AI-DSP	34	(26.1)	22	(21.3)	37.0	29.3	1.412		0.738	2.701	0.326
	Non-AI-DSP	58		53								
rs4680; rs1799732		Met(-);Del(+)		Met(+);Del(-)		Met(-);Del(+)	Met(+);Del(-)					
	AI-DSP	19	(13.9)	40	(36.4)	38.8	31.3	1.393		0.706	2.752	0.375
	Non-AI-DSP	30		88								
rs1799732; rs1800497		Del (+);A1(+)		Del (-); A1(-)		Del (+);A1(+)	Del (-); A1(-)					
	AI-DSP	26	(17.1)	36	(27.5)	42.6	36.7	1.279		0.669	2.449	0.505
	Non-AI-DSP	35		62								
Triple SNP												
rs10770141; rs4680; rs1800497		T(+);Met(-);A1(+)		T(-);Met(+);A1(-)		T(+);Met(-);A1(+)	T(-);Met(+);A1(-)					
	AI-DSP	9	(4.0)	20	(19.0)	64.3	29.9	4.230		1.306	13.619	**0.029**
	Non-AI-DSP	5		47								
rs10770141; rs4680; rs1799732		T(+);Met(-);Del(+)		T(-);Met(+);Del (-)		T(+);Met(-);Del(+)	T(-);Met(+);Del (-)					
	AI-DSP	5	(2.6)	35	(32.1)	55.6	31.0	2.786		0.759	10.202	0.152
	Non-AI-DSP	4		78								
rs10770141; rs1800497; rs1799732		T(+);A1(+);Del(+)		T(-); A1(-);Del(-)		T(+);A1(+);Del(+)	T(-); A1(-);Del(-)					
	AI-DSP	5	(2.8)	33	(24.9)	50.0	37.1	1.697		0.486	5.927	0.501
	Non-AI-DSP	5		56								
rs4680; rs1800497; rs1799732		Met(-);Al(+);Del(+)		Met(+);A1(-);Del(-)		Met(-);Al(+);Del(+)	Met(+);A1(-);Del(-)					
	AI-DSP	11	(7.1)	13	(13.9)	44.0	26.5	2.176		0.803	5.915	0.189
	Non-AI-DSP	14		36								

Abbreviations: SNP, single nucleotide polymorphism; AI-DSP, dopamine supersensitivity psychosis; OR, odds ratio; Met, methionine; Del, deletion.

^a^Comparisons between two groups were performed using the chi-square test.

### Double SNP analyses

In double SNP analyses, there were 6 patterns of SNP combinations (also see Table 2). The numbers and proportions of Ai-DSP(+) patients ranged from 7 [T(+); Del(+)] to 74 [T(-); Del(-)] and from 29.3% [Met(+); A1(-)] to 57.9% [T(+); Met(-)], respectively. An association with increased Ai-DSP(+) was found in those who carried the risk allelic combination of rs10770141 and rs4680, i.e. T(+)/Met(-): 57.9%, OR 2.654, 95%CI 1.036‒6.787, *P* = 0.048. The frequency of this combination was 5.4%. No further association was obtained in double SNP analyses.

### Triple SNP analyses

For triple SNP analyses, there were 4 observed patterns of SNP combinations (also see Table 2). The numbers and proportions of Ai-DSP(+) patients in each pattern ranged from 5 to 35 and from 26.5% to 64.3%, respectively. The T(+)/Met(-)/Del(+) and T(+)/A1(+)/Del(+) combinations were observed in the smallest numbers of Ai-DSP(+) patients: 5 patients for each. Those who carried T(-), Met(+) and Del(-), i.e., non-risk SNPs, made up the largest population, 35. The highest proportion of Ai-DSP(+) was presented by patients with T(+)/Met(-)/A1(+), and carrying this allelic combination was found to be strongly associated with development of Ai-DSP: 64.3%, OR 4.230, 95%CI 1.306‒13.619, *P* = 0.029. This risk combination was found in 4.0% of all the subjects. The lowest Ai-DSP(+) rate was 26.5%, which was found in those who carried Met(+)/A1(-)/Del(-).

## Discussion

In this preliminary case-control study of 357 schizophrenia patients, we investigated the association between combinations of dopaminergic functional SNPs and Ai-DSP. One hundred thirty patients, 36.4%, were diagnosed as having Ai-DSP. There were significant demographic differences in antipsychotic drug exposure and duration of illness, which are possible risk factors for TD, a major criterion for diagnosing Ai-DSP. However, the finding that Ai-DSP(+) patients were taking higher doses of antipsychotics compared to Ai-DSP(-) patients was considered reasonable, since the former were classified as treatment-resistant, which by definition means they required increased antipsychotics to control psychotic symptoms. In addition, the potential inclusion of false negatives, i.e. pre-disease Ai-DSP patients, in the Ai-DSP(-) group cannot be ignored but may have been limited, since the post-hoc analysis showed no significant difference in durations of illness between Ai-DSP(+) with TD and Ai-DSP(-) patients (S2 Table).

Associations of these functional SNPs with schizophrenia have been investigated previously, but the results have not been consistently significant [26–28]. In single SNP analysis, neither allelic nor genotypic association with Ai-DSP was observed. On the other hand, combined SNP analysis demonstrated that Ai-DSP(+) patients comprised significantly higher proportions of the T(+)/Met(-) and T(+)/Met(-)/A1(+) groups. The functional SNPs employed have directional influences on dopaminergic function, and combined analysis of these SNPs may partly indicate innate comprehensive dopaminergic characteristics. Patients with T(+) and Met(-) might be expected to show relatively higher dopamine synthesis and more rapid dopamine degradation, which also suggests that there are more dynamic alterations in dopamine concentrations at the synaptic cleft. In addition, having either Del(+) or A1(+) suggests the potential for lower naïve DRD2 density. If it could be shown that individuals who have these genetic factors are more likely to develop Ai-DSP, these biological characteristics might be related to its pathology.

The concept of Ai-DSP was first reported in the late 1970s [29] and has regained attention recently [2,11,13]. There have been arguments both for and against the idea [30,31]. However, it is true that clinical features of Ai-DSP are sometimes seen in cases of chronic schizophrenia [6,7]. Although several studies have reported specific pharmacotherapeutic approaches as being effective for preventing and/or improving Ai-DSP [11,23,32,33], an improved understanding of the mechanisms underlying Ai-DSP will further contribute to advances in treatment. In particular, identifying schizophrenic patients with higher risks at an early clinical stage would lead to better prognosis. The risk combinations of functional SNPs observed in this study may be useful as new biomarkers in clinical settings.

Although minor allele frequencies (MAFs) are one of the most important factors in our approach, some MAFs vary widely by ethnicity. For example, the T allele of rs10770141 had the lowest MAF, 5.7%, in our study (S1 Table), whereas the 1000 Genome Project reported its frequency as 38.2% in the American population [34]. The other MAFs found in this study were similar to those of the American populations reported by the project (SNP ID in the present study/1000 Genome Project: rs4680, 32.4%/37.8%; rs1800497, 36.3%/31.1%; rs1799732, 17.1%/15.7%) [35–37]. These ethnic influences on MAFs might suggest additional insights into the diversity in the occurrence of DSP and complexity of TRS treatments.

There are some limitations to consider. First, the sample size was not large enough to provide us with sufficient power for more convincing statistical analyses, leaving our study still exploratory. Our hope is that the findings of this preliminary report will bring insight to scientists and provide future chances for further validations. Second, Ai-DSP was diagnosed by descriptions found in the patients’ medical records, which may be inadequate for a correct diagnosis or assessment of other modifiers such as potential biases and cofounders. Third, other genetic polymorphisms related to dopaminergic signals may be involved in Ai-DSP and should be observed. Clearly, prospective large-scale studies including multiethnic subjects are needed.

## Conclusions

We conducted a preliminary and exploratory study to investigate the association of Ai-DSP in schizophrenia with polymorphisms of genes related to dopamine transmission. We found significant associations between Ai-DSP and combinations of the genetic polymorphisms of *TH*, *COMT* and *DRD2*. The findings are important because of their potential to bring new insights to risk prediction and the development of new treatment methods for schizophrenia.

## Supporting information

S1 Table Genotypic distributions and comparison to Hardy-Weinberg equilibrium of all functional SNPs. Abbreviations: SNP, single nucleotide polymorphism; Met, methionine; Del, deletion; Ins, insertion; MAF, minor allele frequency. ^a^The p values were obtained by using chi-square tests and not corrected for multiple testing.
(DOCX)

S2 Table Comparisons of disease and treatment durations among Ai-DSP(+) with/without TD and Ai-DSP(-) groups. Abbreviations: TD, tardive dyskinesia; CP, chlorpromazine; AI-DSP, antipsychotic-induced dopamine supersensitivity psychosis; SD, standard deviation.
(DOCX)
