# Supplementary material for: Vulnerable combinations of functional dopaminergic polymorphisms to late-onset treatment resistant schizophrenia
Source: PLoS One. 2018 Nov 8;13(11):e0207133. doi: 10.1371/journal.pone.0207133 (PMC6224074; doi:10.1371/journal.pone.0207133)
Supplement: S1 Table — Abbreviations: SNP, single nucleotide polymorphism; Met, methionine; Del, deletion; Ins, insertion; MAF, minor allele frequency. aThe p values were obtained by using chi-square tests and not corrected for multiple testing. (DOCX) [file pone.0207133.s001.docx]

S1 Table: Genotypic distributions and comparison to Hardy-Weinberg equilibrium of all functional SNPs

| SNP ID | Subjects | Genotype, N | | | Genotype, % | | | Estimated occurrence  Hardy-Weinberg Equilibrium, % | | | *P* value ^a^ | MAF, % |
| --- | --- | --- | --- | --- | --- | --- | --- | --- | --- | --- | --- | --- |
| ***TH* gene** | | | | | | | | | | | |  |
| rs10770141 |  | T/T | T/C | C/C | T/T | T/C | C/C | T/T | T/C | C/C | 0.16 | 5.7 |
|  | DSP (N=130) | 0 | 19 | 111 | 0 | 14.6 | 85.4 | 0.5 | 13.5 | 85.9 |  |  |
|  | Non-DSP (N=227) | 0 | 22 | 205 | 0 | 9.7 | 90.3 | 0.2 | 9.2 | 90.5 |  |  |
| ***COMT* gene** | | | | | | | | | | | |  |
| rs4680 |  | Met/Met | Val/Met | Val/Val | Met/Met | Val/Met | Val/Val | Met/Met | Val/Met | Val/Val | 0.255 | 32.4 |
|  | DSP (N=128) | 10 | 54 | 64 | 7.8 | 42.2 | 50 | 8.4 | 41.1 | 50.5 |  |  |
|  | Non-DSP (N=224) | 30 | 94 | 100 | 13.4 | 42.0 | 44.6 | 11.8 | 45.1 | 43.1 |  |  |
| ***DRD2* gene** | | | | | | | | | | | |  |
| rs1800497 |  | A1/A1 | A1/A2 | A2/A2 | A1/A1 | A1/A2 | A2/A2 | A1/A1 | A1/A2 | A2/A2 | 0.991 | 36.3 |
|  | DSP (N=130) | 19 | 57 | 54 | 14.6 | 43.8 | 41.5 | 13.4 | 46.4 | 40.3 |  |  |
|  | Non-DSP (N=227) | 32 | 100 | 95 | 14.1 | 44.1 | 41.9 | 13.0 | 46.1 | 40.8 |  |  |
| rs1799732 |  | Del/Del | Del/Ins | Ins/Ins | Del/Del | Del/Ins | Ins/Ins | Del/Del | Del/Ins | Ins/Ins | 0.570 | 17.1 |
|  | DSP (N=130) | 5 | 39 | 86 | 3.8 | 30.0 | 66.2 | 3.6 | 30.6 | 65.9 |  |  |
|  | Non-DSP (N=227) | 5 | 63 | 159 | 2.2 | 27.8 | 70.0 | 2.6 | 27.0 | 70.4 |  |  |

Abbreviations: SNP, single nucleotide polymorphism; Met, methionine; Del, deletion; Ins, insertion; MAF, minor allele frequency

^a^ The p values were obtained by using chi-square tests and not corrected for multiple testing.
