# Supplementary material for: Vulnerable combinations of functional dopaminergic polymorphisms to late-onset treatment resistant schizophrenia
Source: PLoS One. 2018 Nov 8;13(11):e0207133. doi: 10.1371/journal.pone.0207133 (PMC6224074; doi:10.1371/journal.pone.0207133)
Supplement: S2 Table — Abbreviations: TD, tardive dyskinesia; CP, chlorpromazine; AI-DSP, antipsychotic-induced dopamine supersensitivity psychosis; SD, standard deviation. (DOCX) [file pone.0207133.s002.docx]

Table 2: Comparisons of disease and treatment durations among Ai-DSP(+) with/without TD and Ai-DSP(-) groups

|  | Schizophrenia cases | | | | | | *P* values | | | |
| --- | --- | --- | --- | --- | --- | --- | --- | --- | --- | --- |
|  | A | | B | | C | | ANOVA | Tukey | | |
|  | TD(+) Ai-DSP(+)  (*N*=34) | | TD(-) AI-DSP(+)  (*N*=58) | | AI-DSP(-)  (*N*=207) | |  | A vs B | B vs C | A vs C |
| CP equivalent dose, mean (SD), mg | 619.1 | (465.7) | 828.9 | (485.4) | 519.2 | (436.2) | **0.000** | 0.056 | **0.000** | 0.405 |
| Disease duration, mean (SD), years | 28.4 | (16.9) | 33.0 | (13.2) | 23.2 | (15.6) | **0.000** | 0.334 | **0.000** | 0.159 |
| Treatment duration, mean (SD), years | 27.5 | (17.1) | 32.2 | (14.0) | 22.2 | (15.6) | **0.000** | 0.329 | **0.000** | 0.155 |

Abbreviations: TD, tardive dyskinesia; CP, chlorpromazine; AI-DSP, antipsychotic-induced dopamine supersensitivity psychosis; SD, standard deviation
